# KIF15 is involved in development and progression of Burkitt lymphoma

**DOI:** 10.1186/s12935-021-01967-z

**Published:** 2021-05-13

**Authors:** Zhao Wang, Meiting Chen, Xiaojie Fang, Huangming Hong, Yuyi Yao, He Huang

**Affiliations:** 1 Department of Sun Yat-Sen University Cancer Center, State Key Laboratory of Oncology in Southern China, and Collaborative Innovation Center of Cancer Medicine, 651 Dong feng East Road, Guangzhou, 510060 Guangdong China; 2 Department of Medical Oncology, Sun Yat-Sen Memorial Hospital of Sun Yat-Sen University, 107 Yanjiang West Road, Guangzhou, 510120 Guangdong China

**Keywords:** KIF15, BL, Proliferation, Apoptosis, Migration

## Abstract

**Background:**

Burkitt lymphoma (BL) is a highly aggressive, fast-growing B-cell non-Hodgkin's lymphoma, manifested in several subtypes, including sporadic, endemic, and immunodeficiency-related forms, the mechanism of which is still not clear. Abundant evidence reported that KIF15 was involved in the progression of human cancer. The emphasis of this study is to explore the functions of KIF15 in the development of BL.

**Methods:**

Firstly, tumor and normal tissues were collected for detecting expression of KIF15 in BL. Lentivirus-mediated shRNA knockdown of KIF15 was used to construct BL cell model, which was verified by qRT-PCR and Western Blot. The cell proliferation was detected by CCK8 assay, cell apoptosis and cell cycle were measured through flow cytometry. Transwell assay was conducted to detect the migration.

**Results:**

We first found that KIF15 is highly expressed in BL. Knockdown of KIF15 can inhibit proliferation and migration, promote apoptosis and arrest the cell cycle. Moreover, KIF15 is involved in BL cell activity through regulating expression of apoptosis-related proteins (Caspase3, Caspase8, HTRA, IGFBP-6, p53, SMAC, sTNF-R1, TNF-β and Bcl-2) and downstream pathways, such as p-Akt, CCND1, CDK6 and PIK3CA.

**Conclusions:**

These findings justify the search for small molecule inhibitors targeting KIF15 as a novel therapeutic strategy in BL.

**Supplementary Information:**

The online version contains supplementary material available at 10.1186/s12935-021-01967-z.

## Background

Lymphoma is a malignant tumor that originates in the lymphopoietic system [1], which can be classified into non-Hodgkin’s lymphoma (NHL) and Hodgkin's lymphoma (HL) [2]. Depending on the origin of lymphocyte, it can be divided into B cell, T cell and NK cell lymphoma [2, 3]. Burkitt lymphoma (BL) is a highly aggressive B- NHL, which is characterized by the translocation and dysregulation of the *MYC* gene on chromosome 8 and may involve multiple organ systems [4]. It is recognized that the three subtypes of BL (sporadic, endemic, and immunodeficiency-related) have different epidemiology, risk factors, and clinical manifestations [4]. Most BL exhibits considerable heterogeneity in clinical and pathological features [3, 4]. However, the treatment of different types of BL is similar, and chemotherapy is the main treatment method [5, 6]. In addition, the current treatment regimen uses an intensive multi-drug treatment regimen consisting of doxorubicin, an alkylating agent, vincristine, and etoposide, whether short or long [5]. In addition to traditional therapies, a large number of molecular mechanisms have been gradually explored to find effective targets. For example, Bouska et al., revealed that the BCR signaling pathway is a potential therapeutic target in adult-BL [7]. Recently, Mrdenovic et al., proposed DZ-CIS to overcome CIS resistance in aggressive B-cell BLs, and suggested that DZ-CIS could be used to treat relapsed/refractory aggressive BL [8]. Although BL is so far incurable, impressive response rates have been achieved through molecular target drugs. Consequently, understanding the pathogenesis of BL is crucial for identifying potential therapeutic targets and formulating treatment protocols for BL.

KIF15 belongs to the kinesin superfamily, a group of proteins that share highly conserved motor regions [9]. KIF15 (Kinesin-12) is a terminally oriented kinesin that is localized in a mitotic manner to spindle microtubules and chromosomes [10]. During mitosis, Kinesin is controlled spatially and temporally to ensure that it occurs precisely under steady inward and outward forces [11]. However, overexpression of a few mitotic kinases may result in further outward forces that contribute to a series of adverse events, such as anaphase sister chromatid separation, increased axial separation, and eventual unipolar or bipolar or spindle formation [12]. Such events may lead to an unbalanced distribution of DNA, aneuploidy, metastatic and invasive behavior, even cancer [12, 13]. Moreover, it has been demonstrated that KIF15 plays an important role in the development of several types of human cancers such as pancreatic cancer [14], lung adenocarcinoma [15] and breast cancer [16]. Nevertheless, limited data are available regarding KIF15 in BL, especially in terms of its clinicopathological significance and its impact on molecular mechanisms. Herein, we present our results efforts of KIF15 in BL and its clinical relevance and mechanism.

## Materials and methods

### Immunohistochemical staining (IHC)

A total of 150 BL tissue chips were purchased from Xi 'an Alina Biotechnology Co., Ltd. on April 1, 2019 from patients with diffuse B-cell lymphoma. The inclusion and exclusion criteria of the sample were as follows: the inclusion criteria were (1) clinical study of surgical treatment of BL, regardless of language; (2) the age of the subjects was ≥ 18 years old; (3) patients with BL confirmed by histopathology and/or cytology; (4) There was no contraindication of radiotherapy before treatment. The exclusion criteria were (1) system analysis and review; (2) the age of the subjects was less than 18 years old; (3) patients who were not confirmed by histopathology and/or cytology; (4) contraindications of radiotherapy. The normal tissue samples used in this study are the tissues adjacent to the tumor tissues of BL patients as control. The tissue sections were deparaffinized, repaired and blocked with citric acid antigen, they were incubated with KIF15 antibody at 4 °C overnight. After elution with PBS for 5 times, secondary antibody IgG (1: 400, Abcam, USA, # ab6721) was added and incubated at room temperature for 30 min. Tissue sections were subsequently stained with DAB and hematoxylin for visualization. Images were taken and analyzed under photomicroscope.

### Cell culture

The BL cell line Daudi and NAMALWA were obtained from the Cell Bank of the Chinese Academy of Sciences (Shanghai, China). They were cultured in RPMI1640 medium containing 20% fetal bovine serum (FBS) at the atmosphere of 37 °C, 5% CO_2_ and 95% wet air.

### Lentiviral shRNA vector construction and cell transfection

Three RNA interference target sequences (shKIF-1: GCTGAAGTGAAGAGGCTCAAA, shKIF-2: AGGCAGCTAGAATTGGAATCA, shKIF-3: AAGCTCAGAAAGAGCCATGTT) were designed with KIF15 as the template, and the optimal kinetic parameter target was selected for subsequent experiments. Oligo single stranded DNA containing RNA interference target sequences was synthesized and annealed to produce double stranded DNA. It is then directly connected to the digested lentivirus vector BR-V108 (bioscienceres Co. Ltd., Shanghai, China) via its two restriction sites, Age I (NEB, Cat. # R3101L) and EcoR I (NEB, Cat. # R3101L). The ligated products were transferred to the *E. coli* competent cells, and the positive recombinant was identified by PCR and sent to sequencing for verification. The correct positive recombinant was expanded and cultured to obtain high purity plasmid (EndoFree midi Plasmid Kit, TIANGEN, Cat. #DP118-2). Subsequently, 293 T cells were co-transfected with three plasmids (BR-V108, Helper 1.0 and Helper 2.0) to obtain lentivirus. After 48 h the transfected lentivirus was collected for concentration, purification and quality testing, including physical status (color, viscosity), aseptic test and virus titer test. The prepared lentivirus was used to transfect the Daudi and NAMALWA cells. Finally, the expression of green fluorescent protein was observed under a fluorescence microscope and the transfection efficiency was evaluated transfection.

### Quantitative Real-Time-PCR (qRT-PCR)

First, Daudi and NAMALWA cells were collected and RNA was extracted by Trizol (Thermo Fisher Scientific Cat. # 204211) according to the manufacturer’s instructions. Concentration and quality of extracted RNA were determined by Nanodrop 2000/2000C spectrophotometer. The cDNA was obtained by reverse transcription with the Promega M-MLV kit. Finally, qRT-PCR was performed using cDNA as the template and fusing curve was made. The primer as follows, KIF15: 5′-CTCTCACAGTTGAATGTCCTTG-3′, 5′-CTCCTTGTCAGCAGAATGAAG-3′, GAPDH: 5′-TGACTTCAACAGCGACACCCA-3′, 5′-CACCCTGTTGCTGTAGCCAAA-3′.

### Western Blot

Firstly, total proteins of Daudi and NAMALWA cells were extracted and quantified using BCA protein assay kit (Thermo Fisher Scientific, Cat. # A53227). Then proteins were separated via 10% SDS–polyacrylamide gel electrophoresis (SDS-PAGE). Next, samples were transferred to polyvinylidene difluoride (PVDF) membranes at 4 °C. After blocking, membranes were incubated first with primary antibodies (KIF15, Akt, p-Akt, CCND1, CDK6, PIK3CA and GAPDH) (Additional file 1: Table S1) and then with a secondary antibody (Goat Anti-Rabbit, 1:3000, Beyotime, Beijing, China, Cat. # A0208; Goat Anti-Mouse, 1:3000, A0216). Finally, immunoreactions were visualized using Amersham ECL + plusTM Western Blot system and the blots were imaged using a luminescent image analyzer.

### CCK8 assay

Daudi and NAMALWA cells (3000 cells /well) were cultured in 96-well plates with 100 µL/well. After the cells were cultured for two days after laying the plate, 10 µL CCK8 reagent (Sigma, Cat. # 96,992) was added into the pore before the culture was terminated 2 h. After 4 h, the mixed solution was oscillated for 5 min, OD _450 nm_ value was detected by the enzyme-connected immunodetector and the data was recorded for analysis.

### Flow cytometry apoptotic assay

Daudi and NAMALWA cells transfected with lentivirus were inoculated in a 6 cm culture dish for 5 days. Annexin V-APC was added and stained in dark for 15 min. The percentage of cell phase was determined by Flow Cytometry to evaluate the apoptosis rate and the results were analyzed.

### Flow cytometry cell cycle assay

Daudi and NAMALWA cells with shRNA lentivirus were cultured. After then, PBS containing 0.1% BSA was added, then the cell suspension was centrifuged at 200 g for 5 min. Cells were fixed with ethanol, then stained by propidium iodide (PI). The ratio of cells in the G1, S and G2 phases of the KIF15 knockdown group and the control group were detected and analyzed by Flow Cytometry.

### Transwell assay

The chambers were placed in an empty 24-well plate, 100 μL serum-free medium was added to the chamber. Daudi and NAMALWA cells were resuspended with low serum culture medium. Subsequently, the Transwell chamber was removed and washed with PBS. Then methanol was fixed for 30 min and 0.1% crystal violet was stained for 20 min. Finally, the cells under the microscope was observed, photographed and counted.

### Human apoptosis antibody array

The Human Apoptotic Antibody Array Kit (Abcam, USA, Cat. # ab134001) was detected proteins related to the apoptotic signaling pathway. Lentivirus transfected NAMALWA cells were collected and washed with PBS. Each array antibody membrane was blocked with buffer, which incubated overnight at 4 °C. HRP linked streptavidin was added to the membranes. Protein was visualized using ChemiDoc XRS chemiluminescence. The spot density was quantitatively measured by Quantity One software and normalized to *α*-tubulin level.

### Animal xenograft model

Animal experiment was approved by the Ethics committee of Sun Yat sen University Cancer Center in accordance with guidelines and protocols for animal care and protection. BALB/c female nude mice (4 weeks old) were purchased from Beijing Wei Tong Li Hua laboratory animal technology Co., Ltd (Beijing, China). A total of 10 mice were randomly divided into two groups (shCtrl and shKIF15). Adequate NAMALWA cells were counted with a blood cell counting board and finally resuspended with a certain volume of D-hanks. The right forearm armpit of each mouse was subcutaneously injected with 200 μL cells at concentration of 2 × 10^7^ cells/mL. The mice were anesthetized with 0.7% pentobarbital sodium intraperitoneally at a dose of 10 μL/g. Subsequently, the tumor load was evaluated with bioluminescence imaging and analyzed with the IVIS spectral imaging system (emission wavelength 510 nm). Subsequently, tumor size and mouse weight were measured every other day until 10 days after subcutaneous injection. After 42 days, the mice were executed with cervical spine, tumor was removed from the mice. Finally, the tumor was weighed and photographed.

### Ki67 staining

Tumor tissues were sectioned from the sacrificed mice. Afterwards, they were repaired and blocked with the citrate antigen. Antibody Ki67 (1: 200, Abcam, USA, Cat. # ab16667) was added to the shKIF15 or shCtrl, respectively. Subsequently, they were mixed and incubated overnight. PBS elution was performed several times before and after antibody addition. Secondary antibody IgG (1: 400, Abcam, USA, Cat. # ab6721) was added and incubated at room temperature for 30 min. Tissue slices were first stained with DAB, and then with hematoxylin. Images were collected with a photomicroscope and analyzed.

### Statistical analysis

The data were expressed as mean ± SD (n ≥ 3) and analyzed with GraphPad Prism 7.0 software (GraphPad Software Inc., San Diego, CA, USA). The qRT-PCR was analyzed by 2^−∆∆CT^ method. T-test were used to compare the difference. *P* values less than 0.05 were considered statistically significant.

## Results

### KIF15 is upregulated in BL tissues and cells

**Fig. 1:**
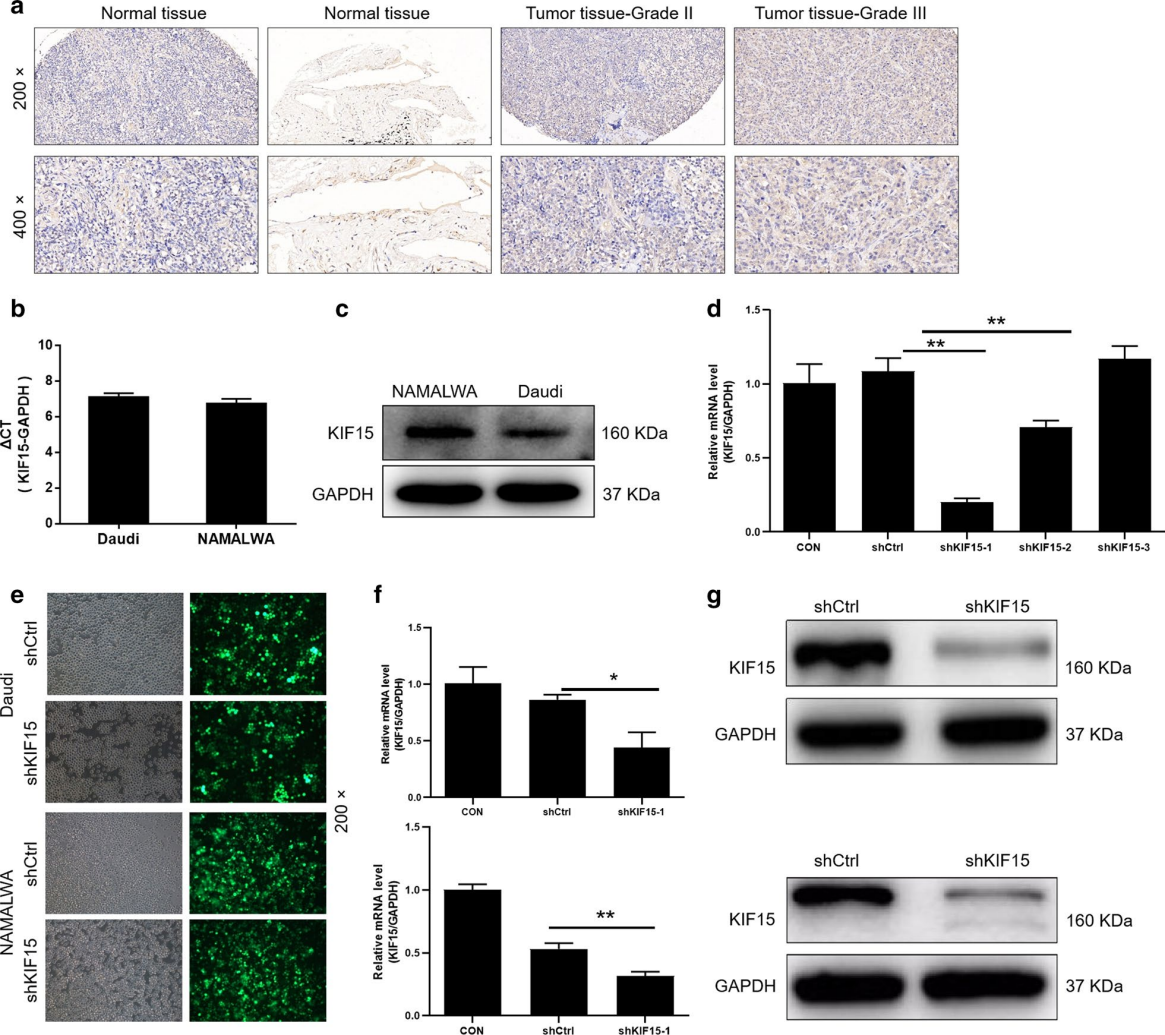
KIF15 is highly expressed in BL and the construction of KIF15 knockdown cell model. **a** IHC staining of KIF15 proteins in BL samples. The expression of KIF15 in the Daudi and NAMALWA detected by qRT-PCR (**b**) and Western Blot (**c**). **d** Screening for effective interference targets by qRT-PCR. **e** Transfection efficiency for Daudi and NAMALWA cells was evaluated by expression of green fluorescent protein 72 h post-infection. The specificity and validity of the lentivirus-mediated shRNA knockdown of KIF15 expression was verified by qRT-PCR (**f**) and Western Blot analysis (**g**). The data were presented as the mean ± SD (n = 3), *P < 0.05, **P < 0.01, ***P < 0.001

According to the IHC analysis (Table 1) (Fig. 1a), the expression of the KIF15 in the tumor tissues of BL was significantly higher than that of the normal tissues (*P* < 0.001). In addition, qRT-PCR results showed that mRNA level of KIF15 was abundantly expressed in BL cells Daudi and NAMALWA (Fig. 1b). Consistently, the protein expression of KIF15 is highly in Daudi and NAMALWA cells (Fig. 1c). The above results indicated that KIF15 is upregulated in BL tissues and cells. Daudi and NAMALWA cells were selected to construct a KIF15 knockdown cell model for subsequent experiments.

**Table 1:** Expression patterns in lymphoma tissues and lymph node tissues revealed in immunohistochemistry analysis

KIF15 Expression	Lymphoma tissues		Lymph node tissues		*P* value
	Cases	Percentage	Cases	Percentage	
Low	49	38.0%	10	100%	< 0.0001
High	80	62.0%	–	–

### Construction of KIF15 knockdown cell models

First of all, according to the results of qRT-PCR (Fig. 1d), ShKIF15-1had the highest efficiency of KIF15 knockdown, so it was used to conduct follow-up experiments (*P* < 0.001). After transfection of shCtrl or shKIF15 for 72 h, the state of the cells was observed under the fluorescence microscope, and the transfection efficiency was above 80% (Fig. 1e). Afterwards, qRT-PCR displayed that the expression of KIF15 in Daudi and NAMALWA cells in shKIF15 group were downregulated by 57.5% (*P* < 0.001) and 58.5% (*P* < 0.01), respectively, compared with that in shCtrl group (Fig. 1f). Similar trend was also observed in Western Blot analysis (Fig. 1g). All the above results indicated that the KIF15 knockdown cell model was constructed successfully.

### Silencing of KIF15 inhibits cell proliferation of BL cells

**Fig. 2:**
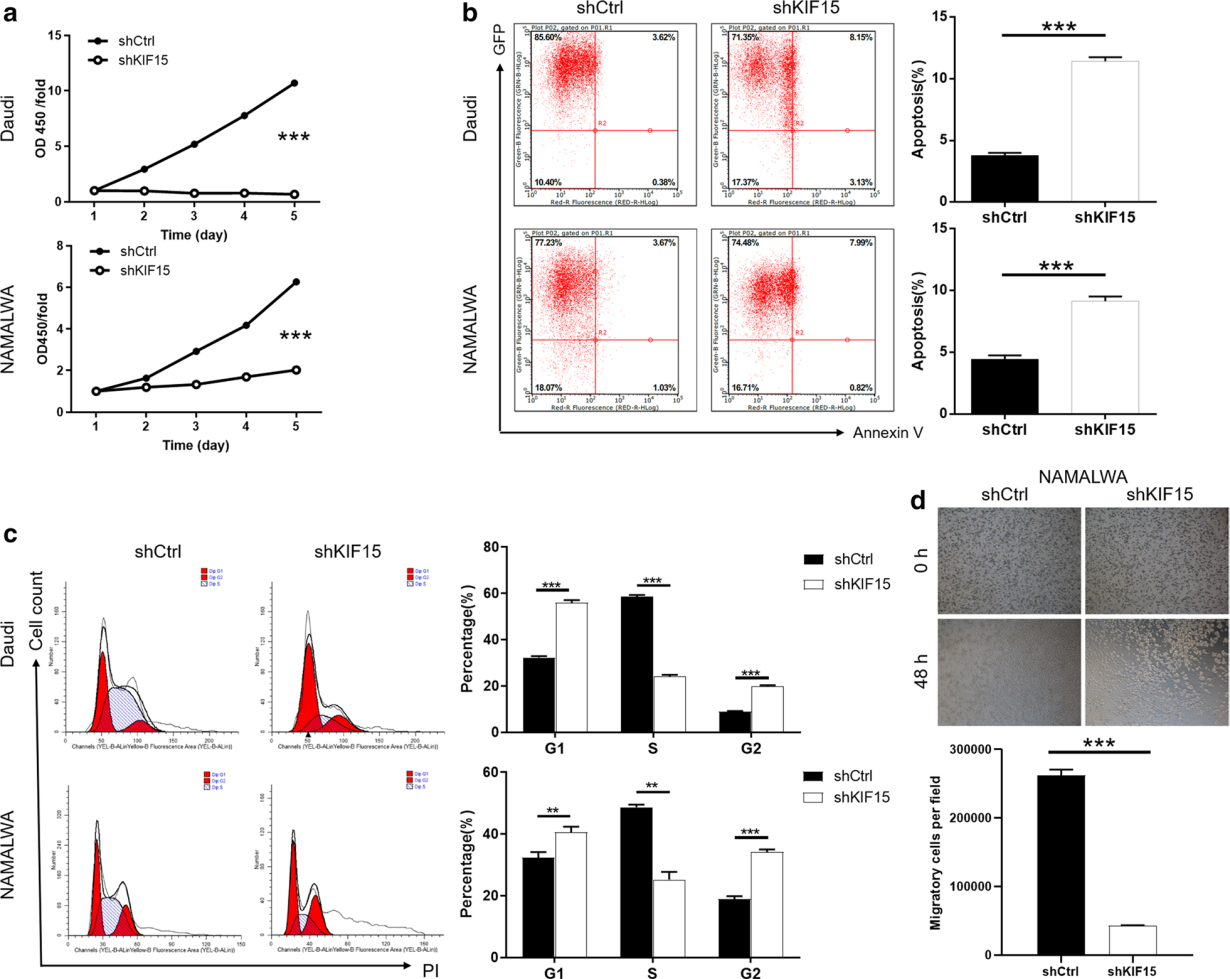
Knockdown of KIF15 inhibits cell proliferation, migration, promotes apoptosis, and regulates EMT-related protein expression in BL cells. **a** Cell proliferation of Daudi and NAMALWA cells with or without knockdown of KIF15 was evaluated by CCK8 assay. **b** Flow cytometry analysis based on Annexin V-APC staining was utilized to detect the percentage of early apoptotic cell for Daudi and NAMALWA cells. **c** Cell cycle was evaluated for Daudi and NAMALWA cells with or without KIF15 knockdown. **d** Cell migration of NAMALWA cells with or without knockdown of KIF15 was evaluated by Transwell assay. The data were expressed as mean ± SD (n = 3), *P < 0.05, **P < 0.01, ***P < 0.001

After the Daudi and NAMALWA cells transfected with shKIF15 or shCtrl the effects of KIF15 on BL cell growth were detected by CCK8 assay. CCK8 results (Fig. 2a) showed that the proliferation rate of BL cell lines in the shKIF15 group were slower than that in the shCtrl group (*P* < 0.001). The data of this study indicated that KIF15 could promote the proliferation of BL cells.

### Silencing of KIF15 induces apoptosis and arrests cell cycle of BL cells

Results of Flow Cytometry apoptotic assay indicated that the percentage of apoptosis in Daudi and NAMALWA cells were promoted by at least 3 folds and 2 folds, respectively (*P* < 0.001) (Fig. 2b). Cell cycle was detected by Flow Cytometry in Daudi and NAMALWA cells of BL. The results, as shown in the Fig. 2c, compared with shCtrl group, the proportion of G2 phase cells in shKIF15 group increased (*P* < 0.001). Thus, the results proved that KIF15 could suppress the apoptosis and arrest cell cycle of BL cells.

### Silencing of KIF15 inhibits migration of BL cells

Cell migration is an important indicator of tumor metastasis, which can be measured by Transwell experiment. Micrograph of Transwell chambers showed that the number of NAMALA cell migration lines in shCtrl group was 6.28 folds than that of the shKIF15 group (*P* < 0.001) (Fig. 2d). Consequently, it can be concluded that KIF15 is essential in promoting the metastasis of BL cells to some extent.

### Exploration of downstream molecular mechanism of KIF15 in BL cells

**Fig. 3:**
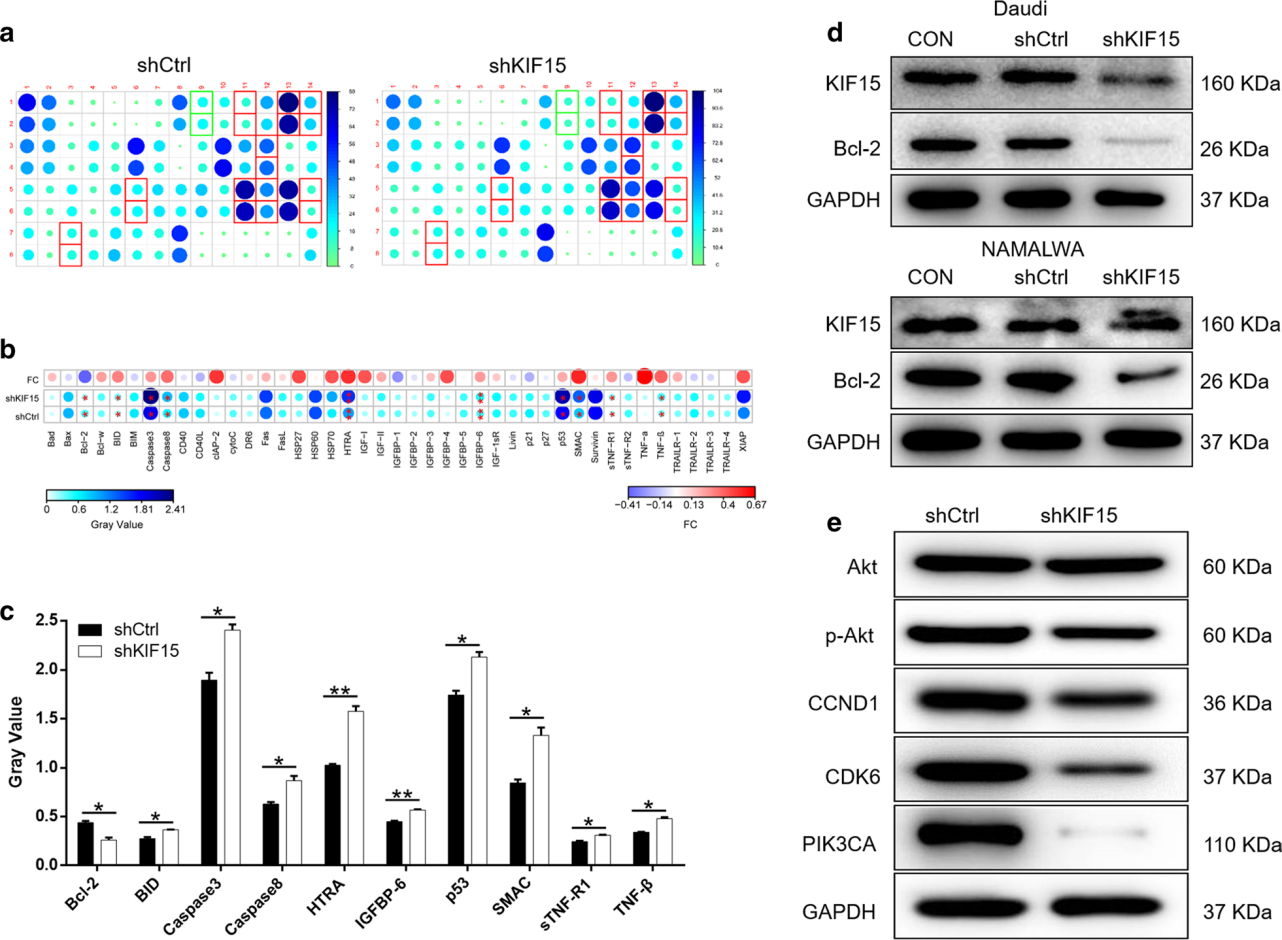
Exploration of downstream molecular mechanism of KIF15 in BL cells. **a**, **b** Human apoptosis antibody array analysis was performed in NAMALWA cells with or without KIF15 knockdown. **c** Densitometry analysis was performed and the gray values of differentially expressed proteins were shown. **d** Expression of apoptosis-related proteins was detected by WB. **e** The expression of target proteins pathways was observed by Western Blot in NAMALWA cells. The data were expressed as mean ± SD (n = 3), *P < 0.05, **P < 0.01, ***P < 0.001

Human Apoptotic Antibody Array was used to analyze the differential expression of 43 proteins between NAMALWA cells in shKIF15 and shCtrl groups. After RNA interference with KIF15 in NAMALWA cells, the related proteins in the human apoptosis signaling pathway were detected. The expression levels of protein BID, Caspase3, Caspase8, HTRA, IGFBP-6, p53, SMAC, sTNF-R1, TNF-β were significantly upregulated, while the expression level of protein Bcl-2 was significantly downregulated (*P* < 0.001) (Fig. 3a–c). In addition, the results of Western Blot indicated that expression of Bcl-2 was downregulated at the protein level (Fig. 3d). The results were consistent with the above data, especially the apoptosis detection. Moreover, Western Blot was also applied to detect the expression of proteins in downstream signaling pathways in NAMALWA cells. Compared with the shCtrl group, there was no significant change in the expression of Akt, but protein expression of p-Akt, CCND1, CDK6 and PIK3CA were downregulated in group shKIF15 (Fig. 3e). Taken together, we can conclude that knockdown of KIF15 affects apoptosis-related proteins and regulates changes in downstream pathways.

### Silencing of KIF15 in BL cells impaired tumorigenesis in vivo

**Fig. 4:**
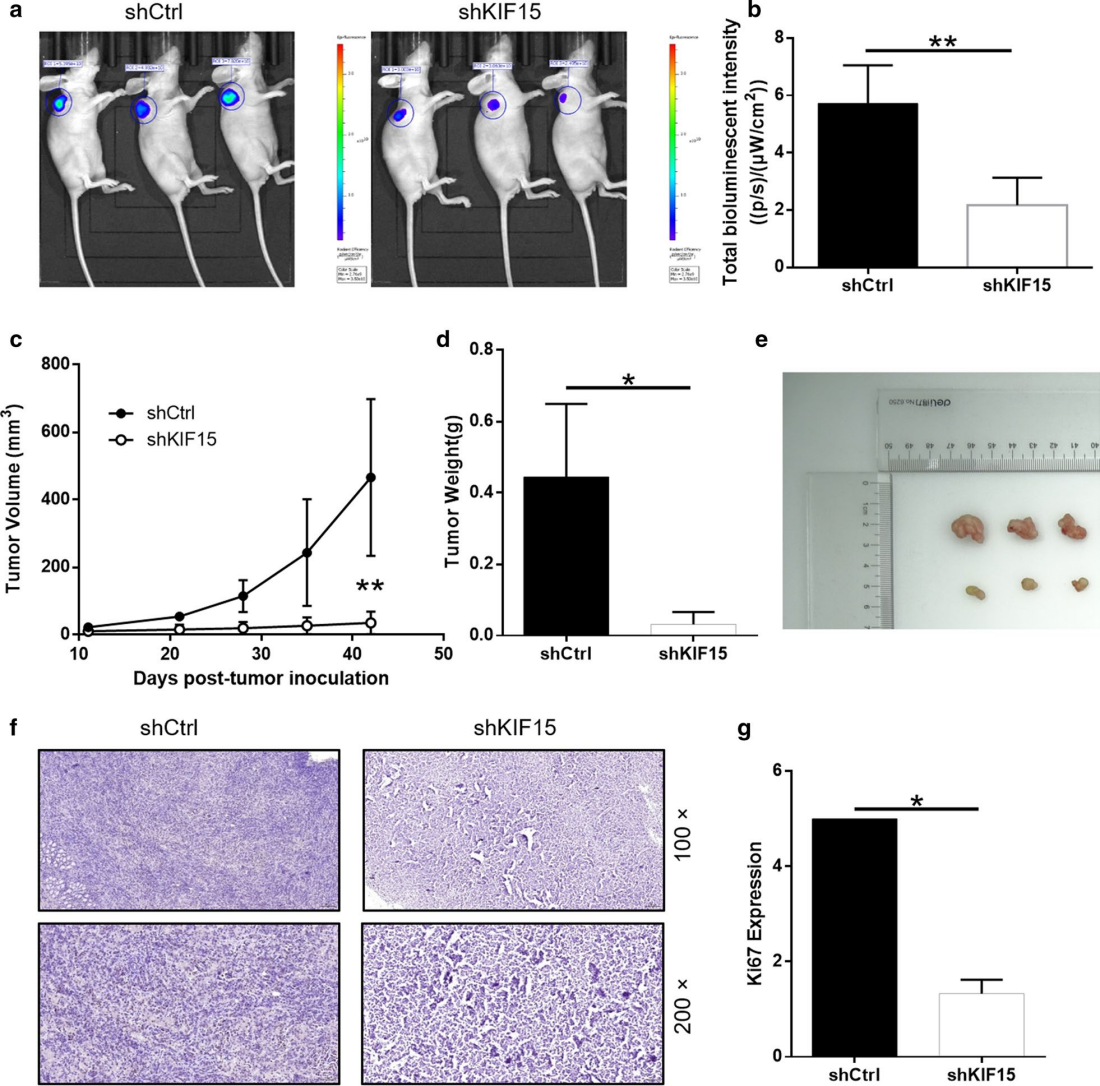
Knockdown of KIF15 inhibits tumor growth in mice xenograft models. **a** The bioluminescence imaging of tumors in shCtrl group and shKIF15 group. **b** The total bioluminescent intensity of tumors in shCtrl group and shKIF15 group. **c** The volume of tumors in shCtrl group and shKIF15 group was measured post-injection. **d** The average weight of tumors in shCtrl group and shKIF15 group. **e** The image of tumors and mice in shCtrl group and shKIF15 group. **f** The Ki67 staining expression of tumor tissues in shCtrl group and shKIF15 group. **g** The Ki67 staining of tumor tissues in shCtrl group and shKIF15 group. The data were expressed as mean ± SD (n = 3), *P < 0.05, **P < 0.01, ***P < 0.001

Animal xenograft model experiment was carried out to verify whether knockdown KIF15 would affect BL cells growth in vivo. The bioluminescence imaging suggested that the intensity of bioluminescence sharply decreased in shKIF15 group than that in shCtrl group (*P* < 0.01) (Fig. 4a, b), the decreased bioluminescence intensity displayed that the tumor growth is weakened. The average volume of tumor of shKIF15 group was sharply decreased in comparison with that of the shCtrl group (*P* < 0.05) (Fig. 4c). What’s more, the average tumor weight of the shKIF15 group was markedly reduced by 0.412 ± 0.171 g than that of the shCtrl group (*P* < 0.05) (Fig. 4d, e). Besides, the Ki67 protein expression in the shKIF15 group was also decreased compared with the shCtrl group (*P* < 0.01) (Fig. 4f, g). The above data proved that knockdown of KIF15 can attenuate the growth of BL cells in vivo.

## Discussion

BL is an aggressive mature B-cell lymphoma that occurs in adults and children and lacks effective treatment options. Previous studies revealed that the survival of BL cells depends on the B cell receptor (BCR) signal, which is an attractive target for drug therapy [14]. In addition, the overexpression of translocation MYC gene is the main cause of BL tumorigenesis. The blockage of JNK pathway can inhibit the expression of immunoglobulin κ and MYC gene, which leads to the inhibition of BL cell proliferation. Accordingly, the JNK pathway was a unique strategy to suppress BL tumorigenesis [15]. Moreover, inhibition of PI3K-Akt-mTOR signal and deregulation of cell cycle and apoptosis through cyclin D3, CDKN2A or TP53 mutations are also considered to be promising targets for BL [16]. Therefore, a thorough understanding of the molecular mechanism of BL is indispensable to identify new potential therapeutic targets.

KIF15 plays critical roles in multiple cancers. Previous studies have shown that KIF15 is overexpressed in a variety of tumors, including pancreatic cancer [17], lung adenocarcinoma, [18] melanoma [19], and breast cancer [13, 21]. In pancreatic cancer, KIF15 promotes the proliferation of cancer cells through the MEK-ERK signaling pathway [17]. Increased KIF15 expression indicates poor prognosis and disordered cell cycle of lung adenocarcinoma [18, 22]. KIF15 also plays a role in promoting the tumorigenicity of melanoma [20]. In breast cancer, the differential expression of the KIF15 was associated with prognosis and promoted cell proliferation also through the MEK-ERK pathway [13, 21]. In addition, KIF15 is being considered as a prognostic marker and a new endocrine therapy target for breast cancer [19]. However, the role of KIF15 in BL has not been clearly defined.

This study is the first to explore the key role of KIF15 in the development and progression of BL. The downregulation of KIF15 inhibits tumor growth by inhibiting proliferation, migration, inducing apoptosis and blocking cell cycle. Moreover, we estimated that KIF15 is involved in the progression of BL by targeting apoptosis-related proteins and regulating the expression of downstream pathways.

During lymphomagenesis, cells encounter a broad range of stress stimuli, including oncogene activation, DNA damage, and oxygen and cytokine deprivation, all of which can elicit an apoptotic response. Complex interactions between pro-apoptotic and anti-apoptotic members of the apoptotic protein family regulate apoptosis [23]. There are several pathways for apoptotic, but the two primary pathways are the endogenous and exogenous pathways. The exogenous pathway is triggered through the binding of death ligands to receptors, while the endogenous pathway is activated by various stimuli such as DNA damage, cell survival factor loss, and are controlled by a series of Bcl family members [24]. For example, pro-apoptotic protein BID is essential for initiating the apoptotic cascade of BL [25, 26]. Mitochondrial apoptosis factor Bax/Bak activates Caspase3 and Caspase7, which triggers apoptosis of NLRP3 inflammatory bodies and Caspase8 driven cells [27]. Studies have shown that IGFB-6 is involved in the regulation of DNA damage repair in cancer cells, inhibiting growth and promoting apoptosis [28, 29]. Tumor suppressor p53 activates the transcription of several genes involved in apoptosis, including BAX, PUMA, and NOXA. Dysregulation of p53 also contributes to lymphoma resistance, since P53 is an important mediator of chemotherapy-induced cell death [28, 30, 31]. In addition, HTRA or SMAC play an important role as a pro-apoptotic protease, inducing cell death mainly by interacting with X-linked apoptotic inhibitor protein (XIAP) [30–33]. The sTNF-R1 and TNF-β play a pivotal role in cellular malignant transformation and is an important mediator of inflammation and apoptosis [34, 35]. On the other hand, Bcl-2 as an anti-apoptotic molecule of the intrinsic apoptotic pathway, inhibits apoptosis by binding to and subsequently inhibiting pro-apoptotic molecules such as Bax and Bak [36]. Evidence continues to reveal that elevated expression of anti-apoptotic Bcl-2 family members (Bcl-2, Bcl-X, Bcl-W, Mcl-1, A1/Bfl-1) is one of the major contributors to lymphomagenesis [25]. In addition, this study supported that knockdown of KIF15 promote apoptosis of BL cells, accompanied by decreased expression of pro-protein BID, Caspase3, Caspase8, HTRA, IGFBP-6, p53, SMAC, sTNF-R1 and TNF-β, while increasing the expression of anti-protein Bcl-2. Therefore, we can conclude that KIF15 is involved in the apoptosis of BL cells through the joint regulation of these apoptosis-related proteins.

The ability of KIF15 knockdown to inhibit the growth of BL cell has prompted us to explore the expression of some classical signaling pathways in cancer. Previous study had declared that blocking PIK3CA can significantly induce lymphoma cell cycle arrest and then induce apoptosis by completely eliminating p-Akt and its downstream target [37]. Giulino-Roth et al., reported that inhibition of PI3K/AKT/mTOR signaling pathway has antitumor activity in BL cells [38]. Mohanty et al., found that CCND1, which belongs to the G1 cell cycle protein, plays an important role in the survival and genomic stability of lymphoma cells [39]. In addition, downregulation of CCND1 and pRb proteins in BL cells is associated with antitumor effects [40]. Jena et al., demonstrated that that CDK6 is required for thymocyte development and for BL induced by activated Akt [41]. However, there is currently no specific study on CDK6 in BL. All in all, results of the present study suggested that the role of KIF15 in regulating cellular processes may involve the expression alteration of p-Akt, CCND1, CDK6, and PIK3CA pathways.

## Conclusions

In summary, this is the first demonstration of the significance of KIF15 in BL. KIF15 is extensively expressed in BL, which can promote proliferation, migration, inhibit apoptosis and arrest cell cycle of BL cells. Moreover, we estimated that KIF15 is involved in BL cell activity through increasing expression of pro-apoptosis proteins, such as Caspase3, Caspase8, HTRA, IGFBP-6, p53, SMAC, sTNF-R1 and TNF-β, meanwhile decreasing expression of anti-apoptosis proteins Bcl-2, and regulating downstream pathways, such as p-Akt, CCND1, CDK6 and PIK3CA. These findings justify the search for small molecule inhibitors targeting KIF15 as a novel therapeutic strategy in BL. Further understanding of cellular and molecular mechanisms will help to identify more potential targets to provide novel therapeutic options for BL patients in the future.

## Supplementary Information

**Additional file 1: Table S1.** The antibody information for the WB.

## Data Availability

Not applicable.
