# Supplementary material for: KIF15 is involved in development and progression of Burkitt lymphoma
Source: Cancer Cell Int. 2021 May 13;21:261. doi: 10.1186/s12935-021-01967-z (PMC8117549; doi:10.1186/s12935-021-01967-z)
Supplement: Supplementary file 1 — Additional file 1: Table S1. The antibody information for the WB. [file 12935_2021_1967_MOESM1_ESM.docx]

| Antibody Name | Protein Size (KDa) | Diluted Multiples | Antibody Source | Company | Number |
| --- | --- | --- | --- | --- | --- |
| KIF15 | 160 | 1:1000 | Rabbit | fine test | FNab04551 |
| Bcl-2 | 26 | 1:500 | Mouse | santa cruz | sc-7382 |
| Akt | 60 | 1:1000 | Rabbit | CST | 4685 |
| p-Akt | 60 | 1:1000 | Rabbit | Bioss | BS-5193R |
| CCND1 | 36 | 1:2000 | Rabbit | CST | 2978 |
| CDK6 | 37 | 1:1000 | Rabbit | Abcam | ab151247 |
| PIK3CA | 110 | 1:1000 | Rabbit | Abcam | ab40776 |
| GAPDH | 37 | 1:3000 | Rabbit | Bioworld | AP0063 |

Table S1. The antibody information for the WB
